# Levels of adherence and factors associated with adherence to option B+ prevention of mother-to-child transmission among pregnant and lactating mothers in selected government health facilities of South Wollo Zone, Amhara Region, northeast Ethiopia, 2016

**DOI:** 10.4178/epih.e2016043

**Published:** 2016-10-13

**Authors:** Delelegn Tsegaye, Leul Deribe, Shambel Wodajo

**Affiliations:** 1 Department of Comprehensive Nursing, College of Medicine and Health Sciences, Wollo University, Dassie, Ethiopia; 2 Department of Nursing and Midwifery, Addis Ababa University College of Health Sciences, Addis Ababa, Ethiopia; 3 Department of Public Health, College of Medicine and Health Sciences, Wollo University, Dassie, Ethiopia

**Keywords:** Medication adherence, Pregnant women, Breastfeeding, Ethiopia

## Abstract

**OBJECTIVES:**

The aim of this study was to measure the levels of adherence and to identify factors associated with adherence to option B+ prevention of mother-to-child transmission (PMTCT) among pregnant and lactating mothers in selected government health facilities of South Wollo Zone, Amhara Region, northeast Ethiopia.

**METHODS:**

An institution-based cross-sectional quantitative study design was employed from March 1, 2016 to April 14, 2016, using a standard structured data collection instrument. A sample of 191 human immunodeficiency virus (HIV)-positive pregnant and lactating mothers who were receiving PMTCT follow-up in the selected health facilities participated in the study. The data were entered using EpiData 3.1 and analyzed using SPSS version 21. Bivariate and multivariate logistic regression analyses were employed to identify factors associated with adherence. The p-values <0.05 and 95% confidence intervals (CIs) were used to identify associations between independent predictors and the outcome variable.

**RESULTS:**

The level of adherence to option B+ PMTCT drugs was 87.9%. Women who received in-hospital treatment, who lived in rural areas, and faced challenges in initiating lifelong option B+ treatment on the same-day that they were diagnosed with HIV were less likely to adhere to the treatment (adjusted odds ratios [95% CI] of 0.3 [0.11 to 0.82], 0.26 [0.1 to 0.73], and 0.08 [0.02 to 0.37], respectively).

**CONCLUSIONS:**

Collaborative efforts of zonal health departments with health facility administrators and counselors are recommended for effective and efficient interventions focusing on hospitals, rural areas, and patients who face challenges on the day of their diagnosis.

## INTRODUCTION

Mother-to-child transmission (MTCT) of human immunodeficiency virus (HIV) remains one of the major sources of HIV infection among young children [1]. In 2013, 3.2 million children under the age of 15 were living with HIV globally, 91% of whom were in sub-Saharan Africa [2]. In order to increase the likelihood of children being born free from HIV, the World Health Organization (WHO) has implemented options A, B, and B+ for the prevention of MTCT (PMTCT) [3].

Option B+ PMTCT involves lifelong antiretroviral therapy (ART) for pregnant and breastfeeding women at the time of HIV diagnosis regardless of CD4 count or clinical stages. A once-daily simplified triple-ART regimen containing tenofovir, lamivudine, and efavirenz (TDF/3TC/EFV) is given [2,4].

Option B+ PMTCT transmits a simple and strong message to mothers (“ART for life”) to promote good adherence and successful ART. The effectiveness of viral load suppression and reducing the MTCT of HIV is highly dependent on adherence to ART [5,6]. According to a WHO report, in women with higher adherence levels to ART, over 80% of their children were protected from HIV, in contrast to a lower percentage among women with a lower adherence rate [7].

Non-adherence increases the risk of virologic failure, maternal HIV disease progression, and the potential development of drug resistance, which may lead to an increased risk of MTCT [8,9]. Low adherence is the main reason for poor treatment outcomes among people receiving ART. In addition to directly affecting mothers’ well-being, poor adherence may hasten the shift from first-line regimens to more expensive second-line regimens at an unnecessarily early stage [10].

Although Ethiopia has implemented many efforts to reduce MTCT, it did not reach the global plan of an MTCT rate <5% [11]. The country faced problems in adherence to ART, as a high percentage of pregnant mothers did not adhere to the full course of lifelong treatment for a variety of reasons [12].

According to a study conducted in the Afar Region of Ethiopia in 2015 on adherence to ART among the non-pregnant and non-breastfeeding population, 19% of clients on ART did not adhere to their drug regimens [13]. A report from the Ministry of Health also indicated that the level of adherence was not good enough to ensure good health outcomes and the reduction of the viral loads resulting from ART [11].

Very few studies have been done in Ethiopia on adherence to option B+ PMTCT among pregnant mothers, and those studies did not include lactating mothers. As option B+ PMTCT is a lifelong treatment, studying the level of adherence among both pregnant and lactating mothers plays an important role in the prevention of new infections. The findings of this study will support the process of controlling MTCT in the study area and in other areas of the country. It will also be beneficial for a range of stakeholders in preventing acquisition of the disease at birth.

## MATERIALS AND METHODS

### Study design and setting

This facility-based cross-sectional study was conducted among HIV-positive pregnant and breastfeeding mothers who were receiving PMTCT follow-up in selected government health facilities in the South Wollo Zone, Amhara Region, northeast Ethiopia from March 1, 2016 to April 14, 2016. The only city in the South Wollo Zone is Dessie, which is located 401 km north of Addis Ababa, the capital city of Ethiopia, and 480 km from Bahir-dar, the capital city of Amhara Region. Two government hospitals and five health centers in Dessie, Kombolcha, and Akesta were selected based on high client flow.

### Sample size determination and sampling technique

The sample size was calculated using a single-population proportion formula with 95% confidence intervals (CIs), a 5% margin of error, and an 87% proportion of adherence estimated based on a study done in the Tigray Region of north Ethiopia [14]. With the incorporation of a 10% non-response rate, the total sample size was calculated to be 191. All study participants meeting the criteria for inclusion were selected until the required sample size was achieved.

HIV-positive pregnant and breastfeeding mothers who had been on ART for at least four weeks and HIV-positive breastfeeding mothers who had sustained breastfeeding until the infant reached six months of age were included in the study. Those who refused to participate were excluded.

### Data collection

A trained interviewer administered a standard structured questionnaire was used [15]. The questionnaire was prepared in English and translated into the local language (Amharic), and then translated back into English by a different person to check the consistency. A pretest was done with 5% of the sample size in Tita health center, the results of which were not included in this study. The data were collected by seven trained nurses who had previous experience in data collection for PMTCT-related studies. The data collection process was closely supervised by one nurse and the principal investigator. The data collectors and supervisor were trained for one day on the objectives, data collection techniques, data quality, and interview techniques for this study. Data consistency and completeness were reviewed on a daily basis.

### Measurements

The dependent variable was adherence to option B+ PMTCT drugs, while the independent variables were socio-demographic variables, service delivery, the stage of the disease and its treatment, and social support. Adherence was measured using pill counting, as well as a self-reporting method adapted from South African experiences [8]. Those mothers who did not miss any antiretroviral (ARV) drugs in the last four weeks prior to the interview and who responded correctly to two or more of the four self-report questions were considered to have good adherence.

A mother was considered to have poor adherence if she respond correctly to less than two of the four self-report measurement questions. Poor adherence was defined using the pill-counting method as missing more than one pill within a month (4 weeks).

If a mother’s adherence levels according to the pill-counting and self-reporting methods were different (good according to one method but poor according to the other); the adherence level was defined using the self-reporting method due to low recall bias in the self-report measurement tool and the fact that it has been recommended for developing countries.

### Data analysis

The collected data were checked daily for completeness and rechecked again by the principal investigator before data entry. Data were entered daily into EpiData 3.1 (EpiData Association, Odense, Denmark), and SPSS version 21.0 (IBM Corp., Armonk, NY, USA) was used for analysis. The entered data were explored for errors and missing values were checked before analysis.

Descriptive statistics were used to summarize the data. Multivariable logistic regression analysis was performed to adjust for possible confounding variables. Variables with a p-value <0.05 in bivariate analysis were entered into the multivariate logistic regression. The p-values <0.05 or 95% CIs not including 1.0 were considered to indicate statistical significance.

### Ethical considerations

Ethical approval of the research proposal was obtained from the ethical review board of Department of Nursing and Midwifery, Addis Ababa University. Permission was obtained from the responsible authorities of each health facility. Detailed explanations of the purpose of the study were given and verbal consent was obtained from all study participants.

## RESULTS

A total of 191 HIV-positive pregnant and breastfeeding mothers were invited to participate in the study, but one mother (0.5%) refused to participate. The mean age of the women was 29.1 years, with a standard deviation of 4.43 years. Of the respondents, 79.5% (n=151) were married. Approximately 49.5% (n=94) of the mothers had completed primary education (grades 1-8) (Table 1).

Of the respondents, 64.2% (n=122) were housewives. The proportion of respondents belonging to the Muslim religion was 55.3% (n=105). Approximately 78.4% of the respondents (n= 149) lived in urban areas. Approximately 55.3% of the women (n=105) traveled more than one hour by walking on foot from their home to a health facility (Table 1).

### Health-related conditions of the respondents

Approximately 88.4% of respondents (n=168) were in WHO clinical stages I and II, and 42.1% of the mothers (n=80) had a CD4 count <350 cells/mm^3^ at the time of admission into the PMTCT B+ program. However, in 22.1% of the HIV-positive pregnant and lactating mothers (n=42), the CD4 count was not measured due to the WHO recommendation to initiate lifelong ART (option B+) irrespective of the CD4 count and clinical stage (Table 2).

Almost all participants (n=189) reported receiving counseling about how to take the option B+ drugs as prescribed. Ninety-seven percent of the respondents (n=185) reported having disclosed their HIV serostatus to others, such as their man partner and family (Table 2).

**Figure 1.**
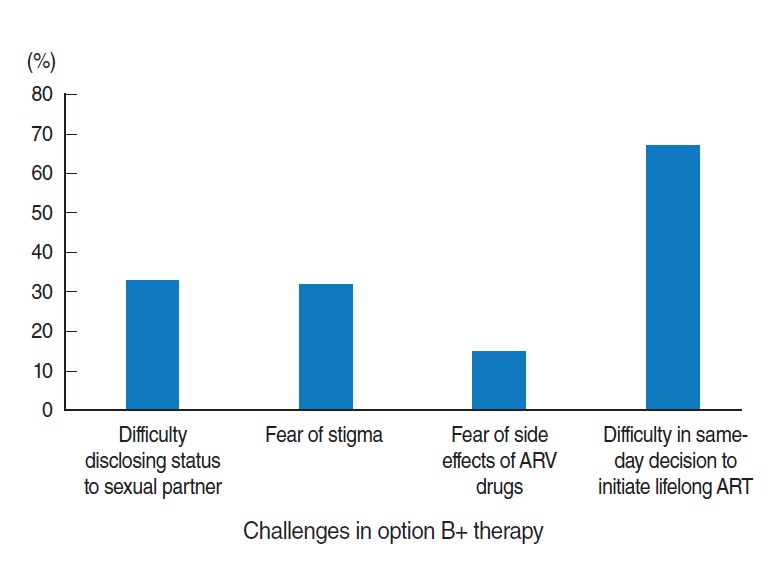
Challenges faced by HIV-positive pregnant and lactating women with regard to the option B+ PMTCT program in selected government health facilities of South Wollo Zone, northeast Ethiopia, 2016 (n=190). The percentages add up to more than 100%. HIV, human immunodeficiency virus; PMTCT, prevention of mother-to-child transmission; ARV, antiretroviral; ART, antiretroviral therapy.

Almost half of the respondents faced at least one challenge in HIV testing and initiating lifelong option B+ treatment on the same-day as their HIV diagnosis (Table 2). Difficulties in decision-making on the same-day as HIV diagnosis were reported by 67% of the participants (Figure 1).

**Figure 2.**
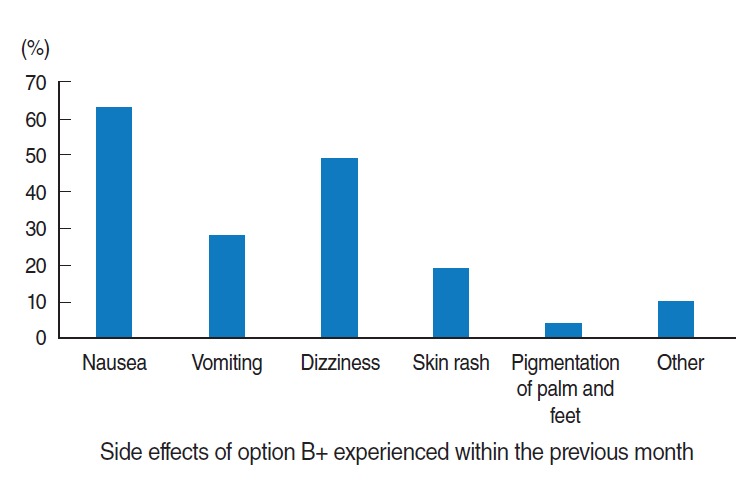
Common option B+ drug side effects experienced by the respondents from selected government health facilities of South Wollo Zone, northeast Ethiopia, 2016 (n=190). The sum of all percentage is greater than 100%. The other side effects were diarrhea and bizarre night-time dreams.

Regarding the side effects of ARV drugs, 58.9% of mothers (n=112) experienced at least one ARV drug side effect. Of those side effects, nausea was the most common, experienced by 56.2% of the women (n=63) (Figure 2).

### Level of adherence to option B+ prevention of mother-to-child transmission drugs

The overall level of adherence measured using both methods was 87.9%. Of the total study participants, 87.9% (n=167) displayed good adherence to the option B+ drugs for PMTCT, whereas 12.1% of mothers (n=23) exhibited poor adherence.

#### Self-reporting

Of the respondents, 96.3% answered “no” to the question “Many patients have trouble taking their ARV doses as prescribed; did you miss any ARV doses in the last 3 days?”. Negative responses were given by 68.4% of the mothers to the question “Do you sometimes find it difficult to remember to take your medication?”.

In response to the 3-day self-reporting question, 92.6% of mothers reported taking all their option B+ drugs correctly within the two days prior to the interview, whereas 90% of mothers reported taking all option B+ drugs within the seven days before the interview.

#### Pill-counting method

The pill-counting method was also used for mothers who brought their ART drug containers and for those who did not by referring to their drug adherence sheet in their medical records. Mothers who missed one or more dose of their TDF/3TC/EVF ARV drugs were considered non-adherent. This was the case for 22 participants (11.6%). One hundred seventy one (90%) participants has reported zero missed pills within 7-day and only nine (4.7%) of them reported as they missed two or more pills within 7-day.

**Figure 3.**
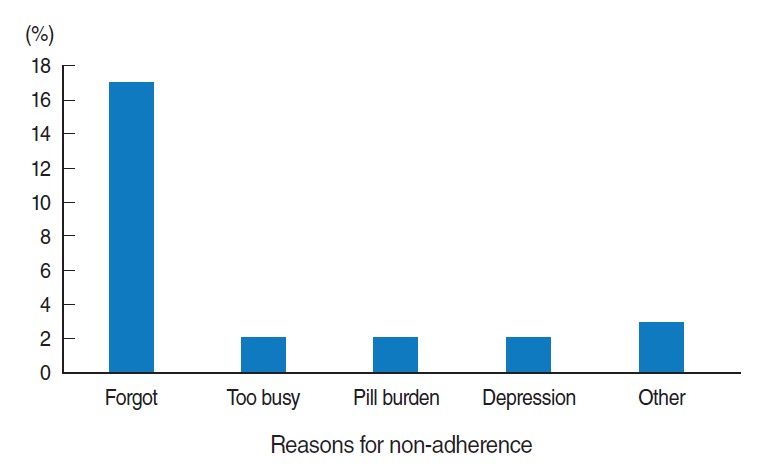
Reasons for non-adherence to option B+ PMTCT drugs among HIV-positive pregnant and lactating mothers in selected government health facilities of South Wollo Zone, northeast Ethiopia, 2016 (n=190). The category of “other” includes side effects of drugs, and lack of support. PMTCT, prevention of mother-to-child treatment; HIV, human immunodeficiency virus.

The main reason for non-adherence to the option B+ drugs was forgetting to take the drugs (77.2%, n=17) (Figure 3).

### Factors associated with adherence to option B+ drugs

Place of residence was the only socio-demographic variable found to have a statistically significant relationship with adherence at the bivariate level. The result of multivariate logistic regression analysis showed that mothers who received option B+ PMTCT services in hospitals were 60% less likely to adhere than those who attended health centers (adjusted odds ratio [aOR], 0.30; 95% CI, 0.11 to 0.80). HIV-positive pregnant and breastfeeding mothers who lived in rural areas were 74% less likely to adhere to option B+ PMTCT drugs than those who lived in urban areas (aOR, 0.26; 95% CI, 0.09 to 0.73). HIV-positive pregnant and breastfeeding mothers who faced challenges in initiating lifelong ART on the day of their HIV diagnosis were also less likely to adhere than those who did not face any such challenges (aOR, 0.08; 95% CI, 0.02 to 0.37) (Table 3).

## DISCUSSION

The level of adherence to option B+ PMTCT drugs among HIV-positive pregnant and breastfeeding women was 87.9%, similar to studies carried out in the Tigray Region of north Ethiopia (87.1%) and Yirgalem Hospital, south Ethiopia (88.7%). This is due to similarities in the socio-demographic characteristics of the populations and the study design used. This level is higher than adherence levels in studies carried out in Sobi Specialist Hospital, Ilorin, Nigeria (73.3%), Dubti Hospital, east Ethiopia (81.1%), and Tikur Anbessa Specialized Hospital, Ethiopia (72.9%) [15-18] among the general population.

The reason for low adherence in other studies may have been that they did not include pregnant and breastfeeding mothers. Pregnant and breastfeeding mothers face many challenges in addition to those faced by the general population, such as nausea, vomiting, and caring for their family and children. Another reason may be that the study design used in Dubti Hospital was retrospective and relied on secondary data [13].

Similarly, studies have been performed to assess the adherence of pregnant and lactating mothers in Chongwe district, Zambia (82.5%), Nnewi, Nigeria (78.3%), and in a meta-analysis (73.5%) [9,16,19]. Socio-demographic factors may explain the discrepancies with our findings.

The option B+ program played a role in improving adherence due to its simplified drug regimen (TDF/3TC/EFV), which is taken in the form of one pill per day. This factor may contribute to the high adherence rate associated with option B+ in comparison with options A or B, which involve taking more pills.

Similarly, the motivation of a mother for her child to be HIV-free may be a reason for good adherence. In this study, 75% of mothers stated that their main reason for adherence was to protect their child from being infected with HIV. Due to this motivation, HIV-positive pregnant and breastfeeding mothers may exhibit high adherence, which is supported by another study carried out in Nigeria [12].

However, in our study, the level of adherence was found to be slightly lower than that reported in a study conducted in Bwaila Hospital, Malawi (91%) [20]. This discrepancy may have been due to the quality of data used for assessing adherence. In that study, they used the pill-counting method based on an electronic medical record system, unlike the method used in our study.

In our study, mothers who received option B+ PMTCT care in hospitals were less likely to be adherent than those who attended health centers. This finding is supported by studies carried out in Zambia and Malawi [19,20]. The potential reasons could include the poor quality of PMTCT services provided in hospitals due to a large number of PMTCT clients. For this reason, health workers could be too busy to provide in-depth adherence counseling.

Mothers who lived in rural areas had lower adherence to option B+ PMTCT drugs than those who lived in urban areas. This is supported by studies carried out in Arba Minch General Hospital, southern Ethiopia and Dubti Hospital, east Ethiopia [13,21].

The reason for this discrepancy is that mothers who lived in rural areas traveled long distances to reach health facilities. In this study, approximately 55.0% of mothers traveled for more than one hour to reach a health facility. Traveling long distances may affect adherence because mothers may get tired and/or face different social problems, meaning that she may miss her appointment. Another reason may be the mothers’ disclosure of their HIV status. In this study, only 26.1% of mothers who lived in rural areas reported having disclosed their HIV status, in sharp contrast to urban residents (73.9%). Disclosure may improve adherence, as suggested by other similar studies [13,14].

HIV-positive pregnant and breastfeeding mothers who faced challenges in testing and initiating option B+ treatment on the same-day as their HIV diagnosis tended to exhibit poor adherence. Very few studies have shown this relationship, but a study carried out among Malawian women described that feeling pressure to initiate ART immediately at the time of HIV diagnosis with little or no support in health-related decision-making was significantly associated with poor adherence [22,23].

In this study, that association appeared logical, since women experiencing challenges in same-day testing and initiating option B+ treatment would seem to be less likely to be confident in the treatment, which may have a negative effect on adherence. In our study, the main challenge they reported facing was difficulty in the early decision to initiate treatment.

To some extent, this study may have been affected by social desirability and recall bias, although those biases were minimized by using strategies such as interviewing in private rooms and using two different adherence measurement methods. The generalizability of our results may be limited to selected health facilities of the South Wollo Zone, as our study participants were only those mothers who received care during the study period.

## Figures and Tables

**Table 1.** Socio-demographic characteristics of participants drawn from northeast Ethiopia, 2016 (n=190)

Variables	Frequency	%
Health facility		
Health centers	122	64.2
Hospitals	68	25.8
Study participants		
Pregnant	94	49.5
Breastfeeding	96	50.5
Age (yr)		
≤29	105	55.3
≥30	85	44.7
Place of residence		
Rural	41	21.6
Urban	149	78.4
Religion		
Muslim	105	55.3
Orthodox	77	40.5
Others	8	4.2
Marital status		
Cohabitating (single, divorced, and widowed)	39	20.5
Married	151	79.5
Respondent’s educational level		
No formal education (cannot read or write)	42	22.1
Grade 1-8	94	49.5
Grade 9-12	37	19.5
Technical/vocational and above	17	8.9
Mother’s occupation		
Housewife (no job)	122	64.2
Private employee	25	13.2
Merchant	21	11.2
Government employee	16	8.4
Others	6	3.0
Male partner’s occupation (n= 151)		
Government employee	41	27.2
Merchant	38	25.2
Day laborer	23	15.2
Private worker	16	10.6
Farmer	22	14.6
Driver	11	7.3
Time taken to reach health facility from home (hr)		
≥1	105	55.3
<1	85	44.7

**Table 2.** Disease- and treatment-related results among pregnant and lactating mothers in selected governmental health facilities of South Wollo Zone, northeast Ethiopia (n=190)

Characteristics	Frequency	%
WHO clinical category at initiation of option B+		
Stage I or II	168	88.4
Stage III or IV	22	11.6
WHO clinical category during the study period		
Treatment stage I	129	67.9
Treatment stage II	61	32.1
Started ART with CD4 count or clinical stage		
Yes	114	60.0
No	76	40.0
CD4 count at initiation of option B+ program (cells/mm^3^)		
< 350	80	42.1
≥350	68	35.8
Not measured during initiation of option B+	42	22.1
Time of initiation of option B+		
During pregnancy	100	52.6
During breastfeeding	15	7.9
Previously started	75	39.5
Challenges faced in same-day diagnosis and initiation of option B+		
Yes	94	49.5
No	96	50.5
Knowledge of HIV status at time of PMTCT enrollment		
Previously known	116	61.1
Newly diagnosed	74	39.0
Received counseling about how to take ART drugs		
Yes	189	99.5
No	1	0.5
No. of pills taken per day (including non-ARV)		
1	87	45.8
2-4	103	54.2
Experienced any ARV side effects		
Yes	112	58.9
No	78	41.1
Regimen changed after side effects		
Yes	16	14.3
No	96	85.7

WHO, World Health Organization; HIV, human immunodeficiency virus; ARV, antiretroviral; ART, antiretroviral therapy; PMTCT, prevention of mother-to-child transmission.

**Table 3.** Bivariate and multivariate logistic regression analysis of factors associated with adherence to option B+ PMTCT among HIV-positive pregnant and breastfeeding women in selected government health facilities of South Wollo Zone, Amhara Region, northeast Ethiopia, 2016

Variables	Adherence level		Crude	Adjusted
	Good	Poor		
Health facility				
Health center	114 (93.4)	8 (6.6)	1.00	1.00
Hospital	53 (77.9)	15 (22.1)	0.25 (0.10, 0.60)	0.30 (0.11, 0.80)^*^
Age (yr)				
≤29	94 (89.5)	11 (10.5)	1.00	1.00
>30	73 (85.9)	12 (14.1)	0.71 (0.29, 1.71)	0.95 (0.33, 2.76)
Place of residence				
Rural	31 (75.6)	10 (24.4)	0.29 (0.12, 0.74)	0.26 (0.09, 0.73)^*^
Urban	136 (91.3)	13 (8.7)	1.00	1.00
Marital status				
Cohabitating	31 (79.5)	8 (20.5)	1.00	1.00
Married	136 (90.1)	15 (9.9)	2.34 (0.91, 6.00)	1.31 (0.43, 3.93)
Educational level				
No formal education	35 (83.3)	7 (16.7)	1.00	1.00
Formal education	132 (89.2)	16 (10.8)	1.65 (0.63, 4.32)	1.63 (0.47, 5.56)
Challenges faced in same-day diagnosis and initiating option B+ treatment				
Yes	73 (77.7)	21 (22.3)	0.07 (0.02, 0.33)	0.08 (0.02, 0.37)^*^
No	94 (97.9)	2 (2.1)	1.00	1.00
Experienced any ARV side effects				
Yes	91 (81.3)	21 (18.8)	1.00	1.00
No	76 (97.4)	2 (2.6)	8.77 (1.20, 38.60)	3.26 (0.65, 16.36)

Values are presented as frequency (%) or odds ratio (95% confidence interval). PMTCT, prevention of mother-to-child transmission; HIV, human immunodeficiency virus; ARV, antiretroviral.

* p<0.05.
